# Alterations in the Temporal Variation and Spatial Distribution of Blood–Brain Barrier Permeability Following Electromagnetic Pulse Radiation: A Study Based on Dynamic Contrast-Enhanced MRI

**DOI:** 10.3390/brainsci15060577

**Published:** 2025-05-27

**Authors:** Kexian Wang, Haoyu Wang, Ji Dong, Li Zhao, Hui Wang, Jing Zhang, Xinping Xu, Binwei Yao, Yunfei Lai, Ruiyun Peng

**Affiliations:** 1 Beijing Institute of Radiation Medicine, Beijing 100850, China; 18652028116@163.com (K.W.); smart106@126.com (H.W.); djtjwj@163.com (J.D.); lillyliz@163.com (L.Z.); wanghui597bj@163.com (H.W.); zhang115614@163.com (J.Z.); xxpbjhd@163.com (X.X.); ybwcsq@163.com (B.Y.); 2 Institute of Biophysics, Chinese Academy of Sciences, Beijing 100101, China

**Keywords:** electromagnetic pulse, dynamic contrast-enhanced magnetic resonance imaging, blood–brain barrier, permeability, rat

## Abstract

Background: Previous studies have suggested that electromagnetic pulse (EMP) can induce openings in the blood–brain barrier (BBB). However, the temporal variation and spatial distribution of BBB permeability after EMP radiation are difficult to assess using conventional histopathological approaches. Dynamic contrast-enhanced magnetic resonance imaging (DCE-MRI) is a valuable tool for the in vivo evaluation of BBB permeability. The main purpose of this study was to investigate the temporal variation and spatial distribution of BBB permeability after EMP radiation in rats using DCE-MRI. Methods: The dose of EMP was estimated through simulations utilizing a digital rat model comprising 16 distinct brain regions. Then, the changes in BBB permeability of the different rat brain regions at different time points (3 h and 24 h) after EMP radiation were evaluated using quantitative DCE-MRI. Furthermore, the spatial difference in BBB permeability was assessed 3 h after exposure. Finally, the dose–effect relationship between the electric field strength and the BBB permeability was also investigated. Results: The results demonstrated that the changes in the values of volume transfer constant (ΔK^trans^) significantly increased in several rat brain regions at 3 h after 400 kV/m EMP radiation. These changes vanished 24 h after exposure. Meanwhile, no significant spatial differences in BBB permeability were observed after EMP radiation. Moreover, Pearson’s correlation analysis showed that there was a significant positive linear relationship between BBB permeability and the electric field strength within an external electric field strength range of 0 to 400 kV/m at 3 h after EMP radiation. Conclusions: EMP radiation can induce a reversible increase in BBB permeability in rats. Moreover, no significant differences in BBB permeability were found across different brain regions. Additionally, the degree of BBB permeability was positively correlated with the regional electric field strength of EMP radiation within an external electric field strength range of 0 to 400 kV/m at 3 h after EMP radiation. These results indicate the promising potential of employing EMP for transient openings in the BBB, which could facilitate clinical pharmacological interventions via drug delivery into the brain.

## 1. Introduction

The blood–brain barrier (BBB) maintains homeostasis of the brain’s internal environment to ensure precise synaptic transmission [1], playing a crucial role in the normal operation of brain functions. On one hand, irreversible alterations in BBB permeability can lead to a variety of central nervous system pathologies [2]. On the other hand, the BBB impedes the entry of therapeutic drugs for brain diseases into brain tissues. Therefore, the reversible alteration of BBB permeability can provide new pathways for the delivery of drugs into the brain [3].

An electromagnetic pulse (EMP), as a kind of short-term high-voltage pulsed electromagnetic radiation with a fast-rising edge and a wide bandwidth [4], has a wide range of applications in the medical, scientific research, and military fields. Previous studies based on histopathological approaches have demonstrated that EMP radiation can lead to alterations in BBB permeability [2,5,6,7], which presents an opportunity for the application of EMP in the treatment of brain disorders.

Based on histopathological approaches, previous studies have shown that BBB permeability exhibits temporal variations [2,5,7] and spatial variations [6] after EMP radiation. However, histopathological approaches require the execution of animals and lack the capacity to provide quantitative physiological metrics. Therefore, it is difficult to investigate BBB permeability for longitudinal intra-subject monitoring and quantitative comparisons using histopathological approaches, such as the detection of dye or tracer extravasation, including Evans blue [5,8], lanthanum nitrate [2,5], and horseradish peroxidase [7].

Dynamic contrast-enhanced magnetic resonance imaging (DCE-MRI) is a non-invasive and radiation-free imaging technique that can provide quantitative insights into tissue-specific physiological characteristics by tracking the temporal evolution of exogenous contrast agent concentration in vivo. This technique enables the derivation of functional biomarkers related to microvascular permeability characteristics and other pathophysiological features. The analysis methods of DCE-MRI mainly include qualitative [9], semi-quantitative [10,11,12], and quantitative [13] analysis methods. Among these, quantitative analysis methods can provide microvascular permeability metrics, including the volume transfer constant (K^trans^) and the extravascular extracellular volume fraction (v_e_) [14]. These parameters have clear physiological significance, independent of the scanner configuration or the magnetic field strength [13]. Furthermore, these parameters exhibit superior reproducibility for longitudinal intra-subject monitoring and cross-sectional inter-subject comparisons [15], demonstrating great advantages and potential for application in both methodological rigor and clinical translatability compared with qualitative or semi-quantitative approaches. Quantitative studies based on quantitative DCE-MRI techniques have focused on the alterations in BBB permeability induced by various pathological states or certain external factors. For example, Xu et al. [16] used quantitative DCE-MRI to study BBB dysfunction in non-human primates with spontaneous type 2 diabetes mellitus. Bergamino et al. [17] investigated the diagnostic utility of DCE-MRI parameters in assessing tumor-induced BBB disruption. Choi et al. [18] mapped spatiotemporal BBB permeability changes following transient cerebral ischemia. Li et al. [19] applied a quantitative DCE-MRI technique to study BBB permeability changes after traumatic brain injury and compared it with the Evans blue (EB) staining method, demonstrating that the disrupted areas in the BBB identified by the two methods mirrored each other. Wu et al. [20] used a quantitative DCE-MRI technique to compare the effects of three microbubble-assisted focused ultrasound methods for inducing BBB openings. Chu et al. [21] investigated the pattern of BBB permeability changes induced by focused ultrasound combined with a microbubble technique and its correlation with the physical parameters of focused ultrasound using a quantitative DCE-MRI technique. Therefore, we anticipate that quantitative DCE-MRI is a suitable method to investigate EMP-induced BBB permeability changes over time, its spatial distribution across the whole brain, and the dose–effect relationship [19].

In this study, we used the quantitative DCE-MRI technique to study the effects of EMP on BBB permeability, thereby quantitatively revealing the patterns of change in BBB permeability over time, the spatial distribution of BBB permeability across the whole brain, and the dose–effect relationship between BBB permeability and radiation dose, with a view to laying a foundation for the use of EMP to promote drug delivery into the brain.

## 2. Materials and Methods

### 2.1. Animals

The animal studies involved in this work were approved by the Institutional Animal Care and Use Committee of the Beijing Institute of Radiation Medicine (Ethics Number: IACUC-DWZX-2020-780). A total of 21 male specified pathogen-free (SPF) Wistar rats, 8 weeks old and weighing 280~330 g, were obtained from the Charles River Laboratory (Beijing, China). The rats were maintained in a constant environment (24 ± 2 °C, 12 h light/dark cycle, 60% relative humidity) with ad libitum access to food and water. The rats were randomly divided into a Sham group (Sham, *n* = 7), a 100 kV/m EMP radiation group (EMP100, *n* = 7), and a 400 kV/m EMP radiation group (EMP400, *n* = 7).

### 2.2. EMP Radiation Setting

**Figure 1:**
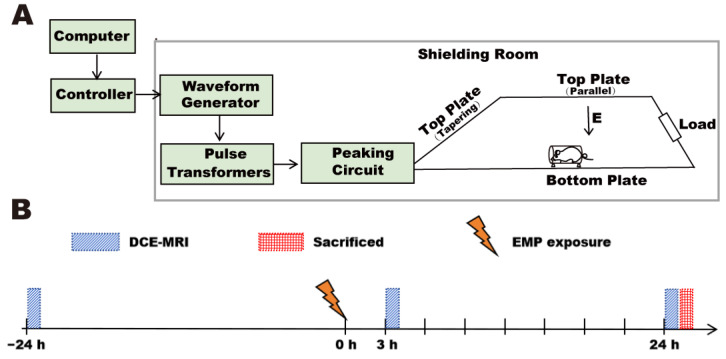
(**A**) The EMP radiation settings; (**B**) the experimental timeline.

A vertical polarized bounded-wave EMP generator developed by the Beijing Institute of Radiation Medicine was implemented in this study (Figure 1A). The whole bodies of the rats were exposed to EMPs with different peak electromagnetic field strengths (peak intensity = 100 ± 25 kV/m and 400 ± 25 kV/m, respectively). The other exposure parameters were as follows: rise time = 5 ns; pulse width = 500 ns; repetition frequency = 1 Hz; number of pulses = 1000. An animal platform and an EMP generator were placed in an electromagnetic shielding room. During exposure, the rats were awake, held in the fixation boxes, and placed on the animal platform. Rats in the Sham group received the same treatment as rats in the EMP-exposed group but with the EMP generator switched off.

### 2.3. Construction of Digital Model of the Rat Brain

**Figure 2:**
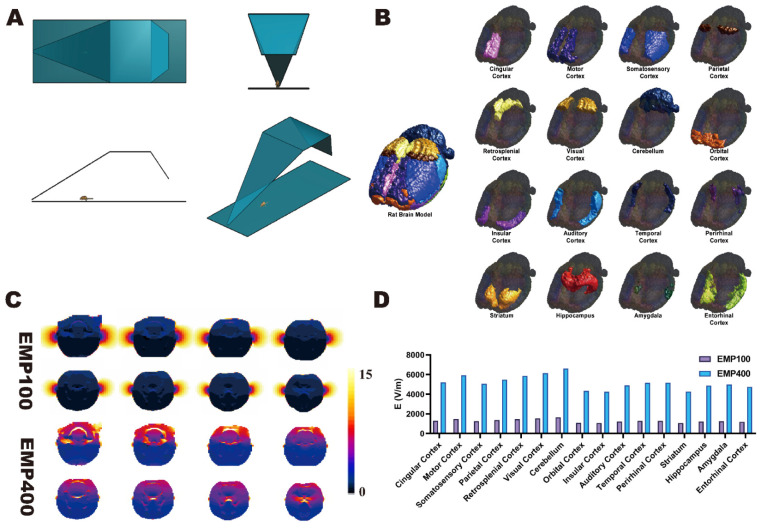
Digital model construction and electric field strength simulation. (**A**) Schematic diagram of the digital model of EMP radiation. The top view (upper left), front view (upper right), side view (lower left), and perspective view (lower right) of the physical model of the EMP radiation source are shown. The rat was positioned in a prone position within the EMP radiation source. (**B**) The digital model of the rat brain with 16 independent brain regions, illustrating the relative positions of the cingulate cortex, motor cortex, somatosensory cortex, parietal cortex, retrosplenial cortex, visual cortex, cerebellum, orbital cortex, insular cortex, auditory cortex, temporal cortex, perirhinal cortex, striatum, hippocampus, amygdala, and entorhinal cortex. (**C**) The simulation results of the electric field distributions in different axial slices of the rats under EMP radiation, with peak external electric field strengths of 100 kV/m and 400 kV/m. The different electric field strengths in rats are indicated by pseudo-colors, and each electric field distribution map ranges from 0 to 15 kV/m. (**D**) The simulation results of the average electric field intensities in various brain regions under EMP radiation with different peak electric field strengths.

Based on the anatomical coordinate information provided by the 5th edition of *The Rat Brain in Stereotaxic Coordinates* (Paxinos and Watson) [22], we constructed a digital model of the rat brain that contained 16 independent brain regions on the basis of the SIGMA brain atlas of the rat [23] (spatial resolution = 0.2 × 0.2 × 0.4 mm; matrix size = 121 × 181 × 51). The model contained the following brain regions: cingulate cortex, motor cortex, somatosensory cortex, parietal cortex, retrosplenial cortex, visual cortex, cerebellum, orbital cortex, insular cortex, auditory cortex, temporal cortex, perirhinal cortex, striatum, hippocampus, amygdala, and entorhinal cortex (Figure 2B). The rat brain model was then imported into the multi-physics simulation software Sim4life V6.0 and fused with a digital rat model containing 51 different tissues based on magnetic resonance images. Thus, a digital rat model containing 16 independent brain regions was established.

### 2.4. Simulation of EMP Radiation Electric Field in Various Brain Regions of the Rat

A dosimetric study of the electric field distribution patterns in the 16 brain regions under EMPs with different peak electromagnetic field strengths was carried out in Sim4life V6.0 using the finite difference time domain (FDTD) method. The corresponding digital model was constructed in the multi-physical parameter simulation software Sim4life V6.0 according to the actual EMP source item device parameters (Figure 2A). The other parameters used in the simulation calculations were as follows: pulse rising edge = 2.4 ns, half-height width = 15.9 ns (these parameters were obtained from the actual EMP waveform).

During the simulation, the free-space EMP peak electric field strengths at the locations of the rat brain were 100 kV/m and 400 kV/m. The grid size of the rat brain region was set to 0.5 × 0.5 × 0.5 mm^3^. For other rat tissues, the grid size was automatically assigned by the simulation software. The tissue electromagnetic parameters (relative permittivity and conductivity) were based on the IT’IS database (version 3.0). A perfectly matched layer (PML) was used as the absorption boundary. Simulations were executed on a dedicated workstation with an Intel Xeon E3-1225 CPU @ 3.2 GHz (Santa Clara, CA, USA), 16 GB DDR4 RAM, and an NVIDIA Quadro K2200 GPU (2 GB VRAM, Santa Clara, CA, USA).

### 2.5. MRI Data Acquisition and Processing

Multi-flip angle T1-weighted images and DCE-MRI images of the rats (Sham group, EMP100 group, EMP400 group) were acquired using an 11.7-T MRI scanner (Preclinical, Bruker, Ettlingen, Germany). All rats in the three groups (Sham, *n* = 7; EMP100, *n* = 7; EMP400, *n* = 7) underwent MRI scans at three time points: at 1 d before EMP radiation and 3 h and 24 h after exposure. The timeline is shown in Figure 1B. During the scanning process, the rats were induced and maintained under anesthesia using isoflurane inhalation, with a gas flow rate ranging from 300 mL/min to 500 mL/min. The induction anesthesia concentration was 3%, and the maintenance anesthesia concentration ranged from 1% to 1.5%. The body temperature was maintained using a water bath holding bed. To minimize motion artifacts during DCE-MRI acquisition, several measures were implemented. The rat’s head was immobilized using a stereotaxic apparatus equipped with bilateral ear bars and an anterior bite bar to minimize motion artifacts during DCE-MRI acquisition. In addition, a respiratory monitoring pad was placed on the chest of the rat to continuously monitor the respiratory rate so as to prevent head movement caused by insufficient anesthesia.

We obtained T1-weighted images using fast low-angle shot (FLASH) pulse sequences. The parameters of the T1-weighted images were as follows: echo time (TE) = 1.1 ms; repetition time (TR) = 42.5 ms; matrix size = 192 × 192; flip angle (FA) = 5°, 10°, 15°, 25°, 30°. We obtained DCE-MRI data using the DCE-MRI sequence. The parameters of the DCE-MRI images were as follows: TE = 1.1 ms; TR = 42.5 ms; FA = 25°; matrix size = 192 × 192; temporal points = 80 scans; imaging interval = 8 s. A bolus dose of gadolinium (Gd-DTPA, Bayer Healthcare Pharmaceuticals, 0.2 mL/kg) was administered via a tail-vein catheter.

Following the MRI data acquisition, data processing was performed using an in-house MATLAB code (MATLAB 2017b; MathWorks, Natick, MA, USA), which was developed by our team. Firstly, according to the method proposed by Tofts et al. [24], the curve of the contrast agent concentration changing with time was calculated voxel by voxel from the DCE-MRI images. Secondly, the two compartment reference region (RR) model proposed by Yankeelov et al. [25] was adopted to estimate the quantitative pharmacokinetic parameters $K_{t}^{trans}$ and $v_{e,t}$ for each voxel. Let $K_{R}^{trans}$ = $K_{t}^{trans}$/$K_{m}^{trans}$ and $v_{e,R}$ = $v_{e,t}$/$v_{e,m}$ be the ratios of the pharmacokinetic parameters between the brain tissue and the reference muscle tissue. According to a previous report [26], the pharmacokinetic parameters of the muscle tissue were taken as $K_{m}^{trans}$ = 0.07 min^−1^ and $v_{e,m}$ = 0.14. The pharmacokinetic parameters of the brain tissue were obtained through simple calculations: $K_{t}^{trans}$ = 0.07 × $K_{R}^{trans}$ and $v_{e,t}$ = 0.14 × $v_{e,R}$. Then, we calculated the voxel-wise K^trans^ and v_e_ values in each rat brain at different time points. Finally, the results of quantitative voxel-wise DCE-MRI analysis were segmented according to the rat brain digital model of 16 brain regions. The average values of the volume transfer constant (K^trans^) and extravascular extracellular volume fraction (v_e_) of the 16 brain regions were calculated to obtain the ROI-wise K^trans^ and v_e_ [13]. The value of K^trans^ represents the rate at which the contrast agent diffuses from the intravascular space into the extravascular extracellular space (EES). The value of v_e_ represents the volume fraction of the extravascular extracellular space (EES) in the tissue per unit volume, with a range between 0 and 1 [27]. To eliminate individual differences among the rats, the changes in the average K^trans^ and v_e_ values (ΔK^trans^ and Δv_e_) of each brain region at 3 h and 24 h after EMP radiation were calculated using the average K^trans^ and v_e_ values of each brain region at 1 d before EMP radiation as the background values.

To mitigate inter-subject variability, we established baseline values of the K^trans^ and ve of each brain region using the K^trans^ and v_e_ values at 1 d before EMP radiation. Alterations in the BBB permeability after EMP radiation were quantified through the changes in the K^trans^ and v_e_ values (ΔK^trans^ and Δv_e_), defined as the relative differences between the values measured at 3 h and 24 h after EMP radiation and their corresponding baseline values.

### 2.6. Statistical Analysis

To investigate the pattern of changes in BBB permeability over time in the various brain regions of rats after EMP radiation, the ΔK^trans^ and Δv_e_ values of each brain region were compared using one-way analysis of variance (one-way ANOVA), followed by post hoc analysis using Tukey’s HSD test. The *p*-values of one-way ANOVA were corrected for multiple comparisons using the Benjamini–Hochberg false discovery rate (FDR) [28]. *p_FDR_* < 0.05 were selected as the significance thresholds in this study. For the post hoc test, compared with the Sham group, * denotes *p* < 0.05, ** denotes *p* < 0.01, and *** denotes *p* < 0.001; compared with the EMP100 group, # denotes *p* < 0.05, and ## denotes *p* < 0.01.

To investigate the spatial distribution of BBB permeability in each brain region of the rats after EMP radiation, the ΔK^trans^ and Δv_e_ values were compared among the brain regions of each group of rats using the paired *t*-test. The *p*-values of the paired *t*-test were corrected for one-way ANOVA using the Benjamini–Hochberg FDR. *p* < 0.05 and *p_FDR_* < 0.05 were selected as the significance thresholds in this study.

To investigate the dose–effect relationship between the electric field strength and the BBB permeability after radiation in the brains of rats exposed to EMP, Pearson’s correlation analysis was carried out between the electric field strength and the corresponding ΔK^trans^ and Δv_e_ values in each brain region. The linear regression equations were obtained through linear regression analysis, with *p* < 0.05 as the criterion of statistical significance.

Statistical analysis was performed using the IBM SPSS Statistics 19 and GraphPad Prism 8.0 software. All results are presented as the mean ± SD in this paper.

## 3. Results

### 3.1. Spatial Distribution of Electric Field in Rat Brains Under EMP Radiation

Based on the constructed digital model of the EMP radiation source (Figure 2A) and the digital rat model with 16 independent brain regions (Figure 2B), the distribution of the average electric field strengths in each brain region under EMP radiation at different external peak electric field strengths (100 kV/m and 400 kV/m) was calculated using FDTD finite element simulation.

As shown in Figure 2C, the electric field distributions in different axial slices of the rats under EMP radiation with different peak electric field strengths of 100 kV/m and 400 kV/m are presented. It can be observed that the electric field strength gradually decreased from the dorsal side to the ventral side of the brain. With 100 kV/m EMP radiation, the value of the electric field strength in the brain ranged from 1064.9675 V/m to 1651.5725 V/m, with an average of 1296.0523 V/m. With 400 kV/m EMP radiation, the value of the electric field strength in the brain ranged from 4259.8700 V/m to 6606.2900 V/m, with an average of 5184.2093 V/m.

The results of the dosimetry study show that as the external electric field strengths of the EMP radiation increased, the electric field strength in the brains of the rats also increased (Figure 2D). Under EMP radiation with the same external electric field strength, the electric field strength in the brains of rats gradually decreased from the dorsal side to the ventral side.

### 3.2. Temporal Variation in BBB Permeability of Rat Brains After EMP Radiation

DCE-MRI was used to study the temporal variation pattern of the BBB permeability in rats at 3 h and 24 h after EMP radiation. One-way ANOVA, followed by Tukey’s HSD post hoc tests, was used to compare the ΔK^trans^ and Δv_e_ in each brain region of the rats among the Sham, EMP100, and EMP400 groups. The *p*-values of the one-way ANOVA were corrected using the Benjamini–Hochberg false discovery rate (FDR) [28].

**Figure 3:**
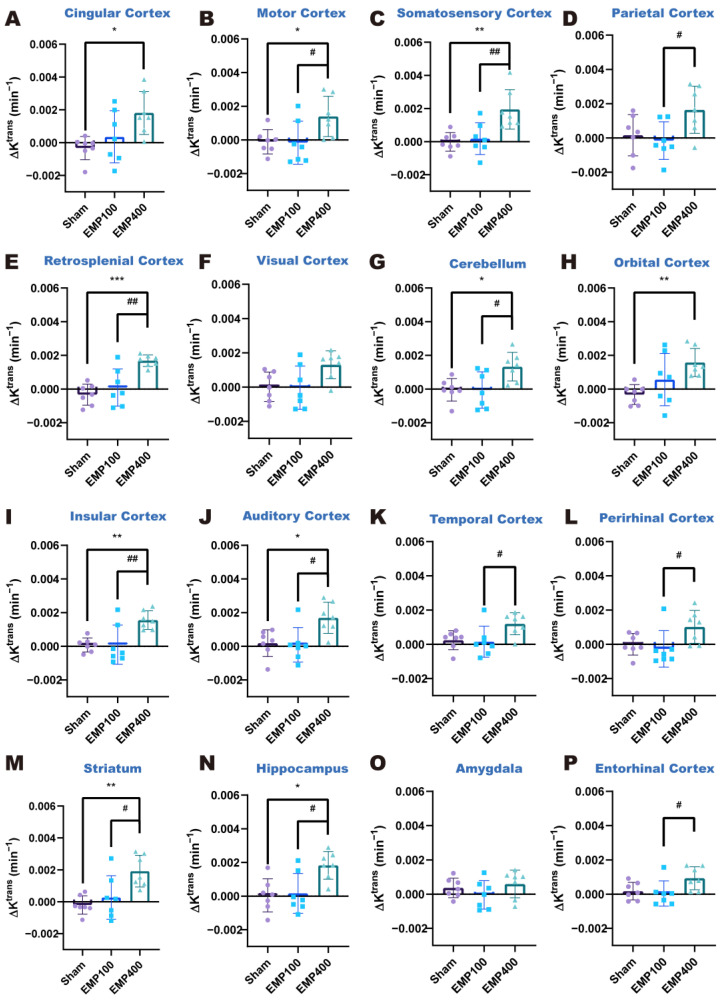
Comparison of the ΔK^trans^ values in each brain region of the rats among the Sham, EMP100, and EMP400 groups at 3 h after EMP radiation. (**A**–**P**) Comparisons of the ΔK^trans^ values in the 16 brain regions of the rats 3 h after EMP radiation, respectively. One-way ANOVA, followed by Tukey’s HSD post hoc tests, was used to compare the ΔK^trans^ in each brain region of the rats among the Sham, EMP100, and EMP400 groups. The *p*-values of one-way ANOVA were adjusted using the Benjamini–Hochberg FDR. For the post hoc test, compared with the Sham group, * indicates *p* < 0.05, ** indicates *p* < 0.01, and *** indicates *p* < 0.001; compared with the EMP100 group, ^#^ indicates *p* < 0.05, and ^##^ indicates *p* < 0.01.

**Figure 4:**
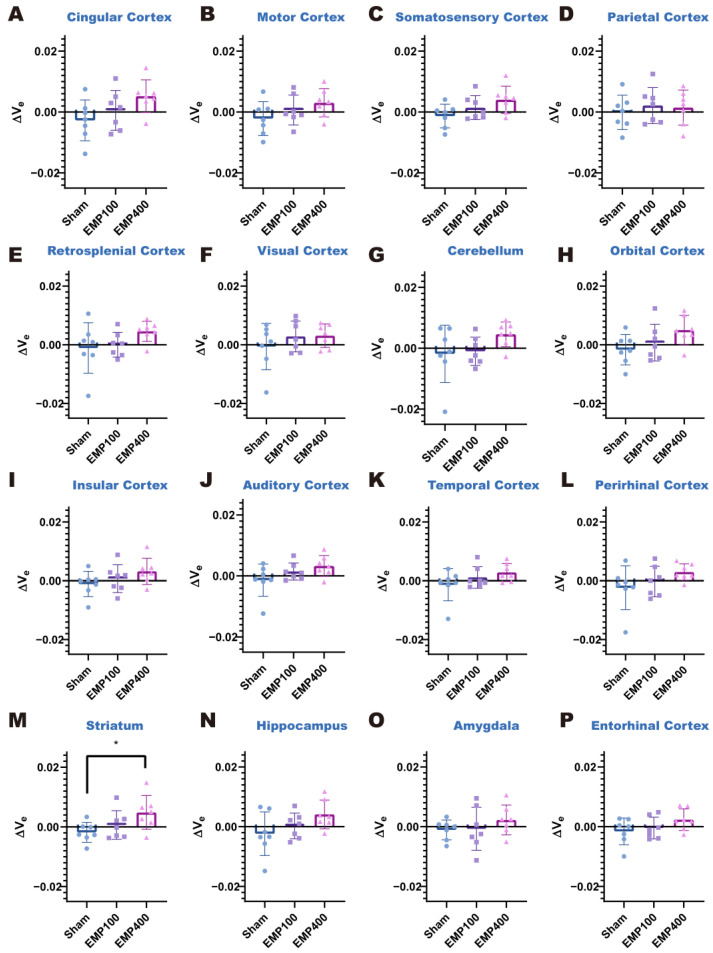
Comparison of the Δv_e_ values in each brain region of the rats among the Sham, EMP100, and EMP400 groups 3 h after EMP radiation. (**A**–**P**) Comparisons of the Δv_e_ values in the 16 brain regions of the rats 3 h after EMP radiation, respectively. One-way ANOVA, followed by Tukey’s HSD post hoc tests, was used to compare the Δv_e_ in each brain region of the rats among the Sham, EMP100, and EMP400 groups. The *p*-values of one-way ANOVA were adjusted using the Benjamini–Hochberg FDR. For the post hoc test, compared with the Sham group, * indicates *p* < 0.05.

We first investigated the changes in BBB permeability in the rats 3 h after EMP radiation. As shown in Figure 3 and Figure 4, at 3 h after 400 kV/m EMP radiation, the cingulate cortex, motor cortex, somatosensory cortex, retrosplenial cortex, cerebellum, orbital cortex, insular cortex, auditory cortex, striatum, and hippocampus of the rats were affected by EMP radiation. Specifically, one-way ANOVA corrected by FDR showed significant differences in ΔK^trans^ in 14 brain regions among three groups, including the cingulate cortex (*p_FDR_* = 0.0276), motor cortex (*p_FDR_* = 0.0352), somatosensory cortex (*p_FDR_* = 0.0120), parietal cortex (*p_FDR_* = 0.0405), retrosplenial cortex (*p_FDR_* = 0.0032), visual cortex (*p_FDR_* = 0.0428), cerebellum (*p_FDR_* = 0.0266), orbital cortex (*p_FDR_* = 0.0266), insular cortex (*p_FDR_* = 0.0120), auditory cortex (*p_FDR_* = 0.0179), temporal cortex (*p_FDR_* = 0.0352), perirhinal cortex (*p_FDR_* = 0.0428), striatum (*p_FDR_* = 0.0120), hippocampus (*p_FDR_* = 0.0179), and entorhinal cortex (*p_FDR_* = 0.0428) among the three groups. Then, according to the results of the post hoc multiple comparisons, compared with the Sham group, the ΔK^trans^ values increased in the cingulate cortex, motor cortex, somatosensory cortex, retrosplenial cortex, cerebellum, orbital cortex, insular cortex, auditory cortex, striatum, and hippocampus of the rats in the EMP400 group. However, there were no significant changes in the rats of the EMP100 group. In addition, one-way ANOVA corrected by FDR showed no significant differences in the Δv_e_ among the three groups (*p_FDR_* > 0.05). These results suggest that BBB permeability increased in the rats 3 h after 400 kV/m EMP radiation, while the unchanged Δv_e_ suggests preserved extracellular space volume in the brain regions.

**Figure 5:**
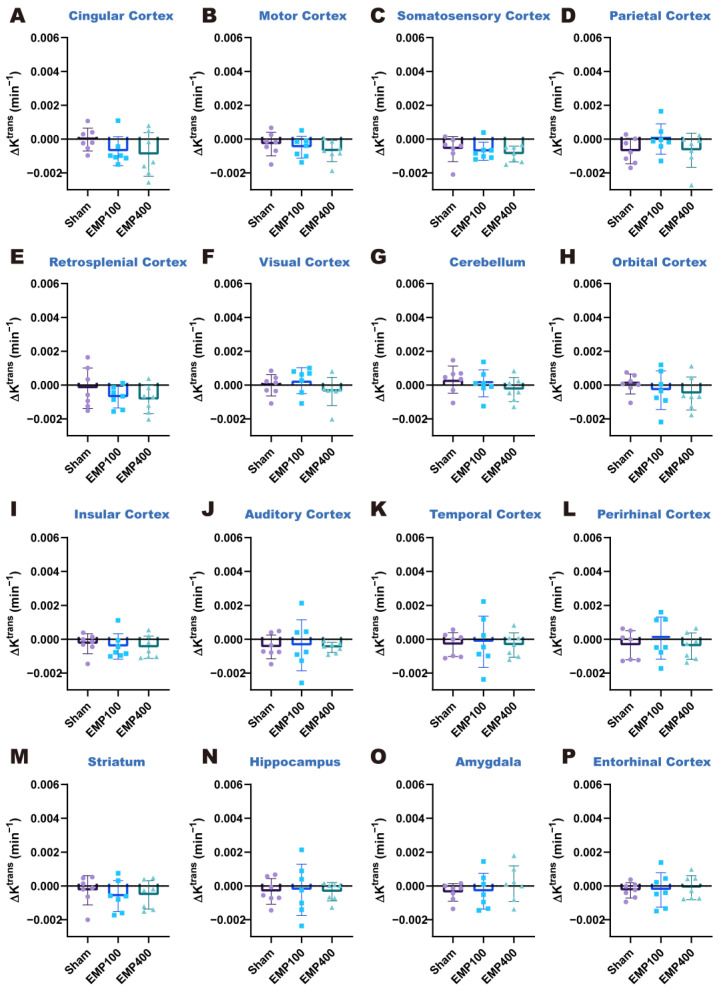
Comparison of the ΔK^trans^ values in each brain region of the rats among the Sham, EMP100, and EMP400 groups 24 h after EMP radiation. (**A**–**P**) Comparisons of the ΔK^trans^ values in the 16 brain regions of the rats 24 h after EMP radiation, respectively. One-way ANOVA, followed by Tukey’s HSD post hoc tests, was used to compare the ΔK^trans^ in each brain region of the rats among the Sham, EMP100, and EMP400 groups. The *p*-values of one-way ANOVA were adjusted using the Benjamini–Hochberg FDR.

**Figure 6:**
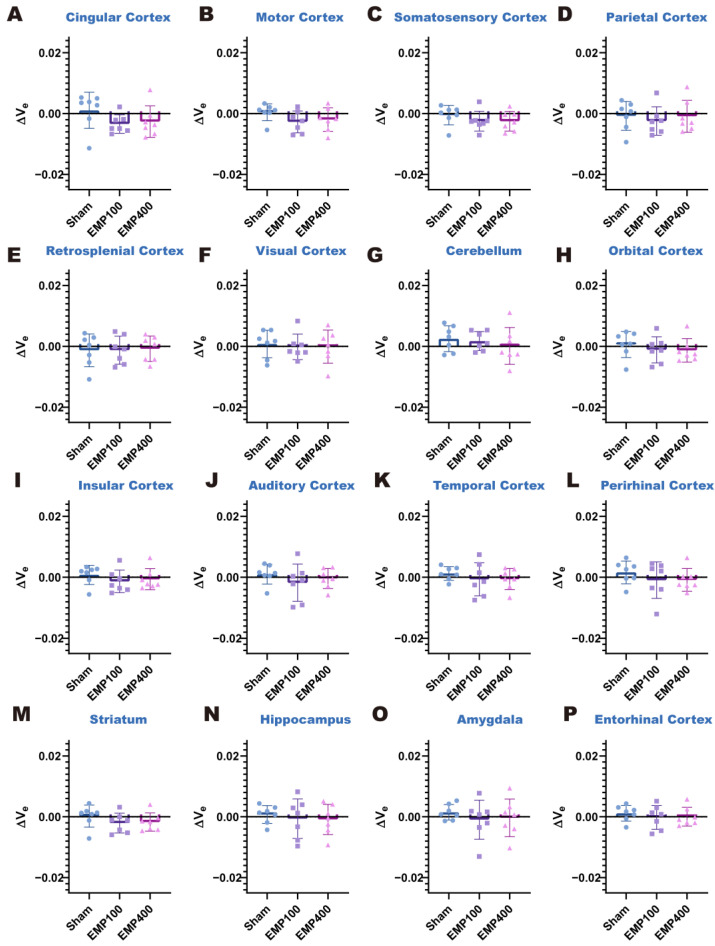
Comparison of the Δv_e_ values in each brain region of the rats among the Sham, EMP100, and EMP400 group 24 h after EMP radiation. (**A**–**P**) Comparisons of the Δv_e_ values in the 16 brain regions of the rats 24 h after EMP radiation, respectively. One-way ANOVA, followed by Tukey’s HSD post hoc tests, was used to compare the Δv_e_ in each brain region of the rats among the Sham, EMP100, and EMP400 groups. The *p*-values of one-way ANOVA were adjusted using the Benjamini–Hochberg FDR.

Then, we investigated the changes in BBB permeability in the rats 24 h after EMP radiation. As shown in Figure 5 and Figure 6, there were no statistically significant differences in the values of ΔK^trans^ and Δv_e_ among the three groups (*p_FDR_* > 0.05). These results suggest that the BBB permeability of the rats recovered 24 h after 400 kV/m EMP radiation.

Taken together, these results suggest that BBB permeability increased in the rats 3 h after being exposed to 400 kV/m EMP radiation. This adverse effect was mitigated 24 h after the exposure. Moreover, 100 kV/m EMP radiation showed no significant effects on BBB permeability either at 3 h or 24 h after the exposure.

### 3.3. Spatial Distribution of BBB Permeability in Rat Brains Under EMP Radiation

Then, we investigated the spatial distribution of BBB permeability in the rats 3 h after EMP radiation at different field strengths (100 kV/m, 400 kV/m). The ΔK^trans^ and Δv_e_ values in each brain region of the rats in different groups (Sham, EMP100, and EMP400) 3 h after EMP radiation were calculated. Paired *t*-tests were used to compare the ΔK^trans^ and Δv_e_ among the different brain regions in each group. The *p*-values of the paired *t*-tests were corrected using the Benjamini–Hochberg FDR [28].

**Figure 7:**
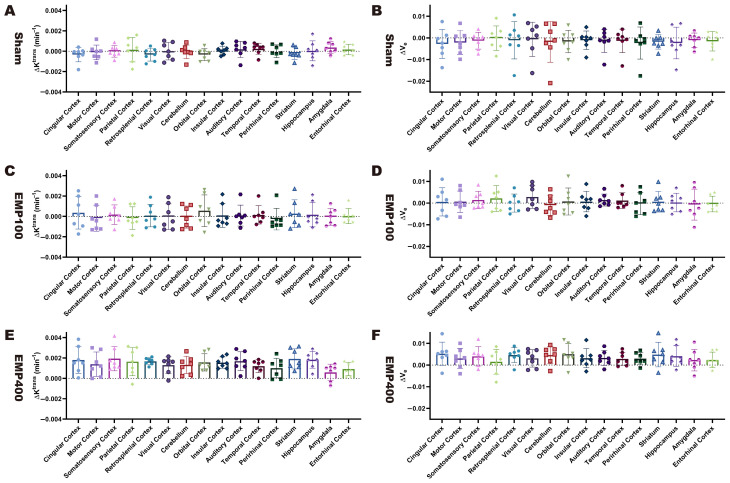
Comparison of the ΔK^trans^ and Δv_e_ values in the different brain regions of the rats 3 h after EMP irradiation. (**A**,**C**,**E**) show comparisons of the ΔK^trans^ values 3 h after EMP radiation in the different brain regions of the rats among the Sham group, EMP100 group, and EMP400 group, respectively. (**B**,**D**,**F**) show comparisons of the Δv_e_ values 3 h after EMP radiation in the different brain regions of the rats among the Sham group, EMP100 group, and EMP400 group, respectively. Paired *t*-tests were used to compare the ΔKtrans and Δve among the different brain regions in each group. The *p*-values of the paired *t*-tests were adjusted using the Benjamini–Hochberg FDR.

Figure 7A,C,E show comparisons of the ΔK^trans^ values among the different brain regions of the rats in the Sham, EMP100, and EMP400 groups, respectively. Paired *t*-tests corrected by FDR showed no statistically significant differences in the ΔK^trans^ values between the pairwise brain regions of the rats across all groups (*p_FDR_* > 0.05). These results indicate that there were no significant differences in the spatial distribution of BBB permeability after EMP radiation. Figure 7B,D,F show comparisons of the Δv_e_ values among the different brain regions of rats in the Sham, EMP100, and EMP400 groups, respectively. Paired *t*-tests corrected by FDR showed no statistically significant differences in the Δv_e_ values between the pairwise brain regions of rats across all groups (*p_FDR_* > 0.05). These results indicate that there were no significant differences in the spatial distribution of the extracellular space volume after EMP radiation.

Taken together, these results suggest that no significant spatial differences in BBB permeability were observed after EMP radiation.

### 3.4. Dose–Effect Relationship Analysis of Electric Field Strength of EMP Radiation and BBB Permeability in Rat Brain

In order to study the dose–effect relationship between the electric field strength and BBB permeability in the rat brains, Pearson’s correlation analysis was carried out to investigate the relationship between the regional electric field strength in the rat brain (E) and the corresponding ΔK^trans^ and Δv_e_ values of all brain regions in the rats at 3 h after EMP radiation. The linear regression equations were obtained using linear regression analysis.

**Figure 8:**
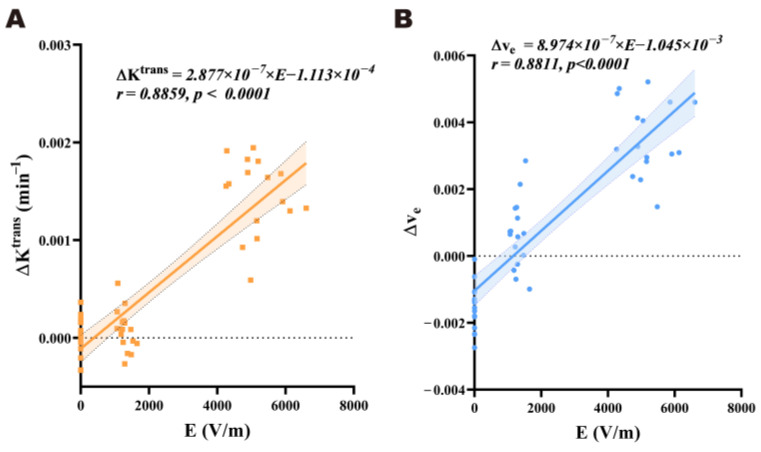
Results of the linear regression analysis between the regional electric field strength in the rat brain E in the rat brain and the ΔK^trans^ value and Δv_e_ value 3 h after EMP radiation. (**A**) The linear relationship between the E value and the corresponding ΔK^trans^ value within an external electric field strength range of 0 to 400 kV/m at 3 h after EMP radiation (*r* = 0.8859, *p* < 0.0001). (**B**) The linear relationship between the E and the corresponding Δv_e_ value within an external electric field strength range of 0 to 400 kV/m at 3 h after EMP radiation (*r* = 0.8811, *p* < 0.0001). Pearson’s correlation analysis was carried out to investigate the relationship between the E values and the corresponding ΔK^trans^ and Δv_e_ values of all brain regions in the rats. The linear regression equations were obtained by linear regression analysis. E represents the regional electric field strength in the rat brain.

As shown in Figure 8A, there was a significant positive linear relationship between the electric field strength (E) and the corresponding ΔK^trans^ value in each brain region within an external electric field strength range of 0 to 400 kV/m at 3 h after EMP radiation (*r* = 0.8859, *p* < 0.0001). The linear regression equation used was Equation (1). As shown in Figure 8B, there was also a significant positive linear relationship between the E and the corresponding Δv_e_ value in each brain region within an external electric field strength range of 0 to 400 kV/m at 3 h after EMP radiation (*r* = 0.8811, *p* < 0.0001). The linear regression equation used was Equation (2).

(1) $${\Delta K}^{trans}=2.877\times 10^{-7}\times E-1.113\times 10^{-4}$$

(2) $$\Delta v_{e}=8.974\times 10^{-7}\times E-1.045\times 10^{-3}$$

Then, we performed a linear regression fitting between the regional electric field strength and the regional BBB permeability within the EMP400 group, as there was no significant difference in either the ΔK^trans^ or Δv_e_ between the Sham and the EMP100 groups. When the external electric field strength was 400 kV/m, the linear relationship between the E and the corresponding ΔK^trans^ (*r* = −0.1513, *p* = 0.5759) or Δv_e_ (*r* = −0.0546, *p* = 0.8409) across the different brain regions was not observed (Supplementary Figure S1).

The above results suggest that there was a significant positive correlation between BBB permeability and the regional electric field strength in the rat brain within an external electric field strength range of 0 to 400 kV/m at 3 h after EMP radiation. However, when the external electric field strength was 400 kV/m, this relationship between the regional electric field strength and BBB permeability across the different brain regions was not observed.

## 4. Discussion

In this study, we first constructed a digital rat model containing 16 independent brain regions and performed an EMP radiation dose simulation to obtain the spatial distribution of the electric field strength across the whole brain under EMP radiation. Then, we utilized quantitative DCE-MRI techniques to investigate the following: (1) changes in BBB permeability over time (3 h and 24 h after radiation compared with 1 d before radiation); (2) the spatial distribution patterns of the effects of EMP radiation at different peak electric field strengths (0 kV/m, 100 kV/m, and 400 kV/m) on the BBB permeability of rats across the whole brain; and (3) the dose–effect relationship between the electric field strength and the changes in the permeability of the BBB in different brain regions.

Exploring the relationship between the EMP radiation dose and structural and functional damage to the brain provides a basis for studying the biological effects of EMP radiation on the brain. Recently, a general rat model constructed using MRI has been widely used due to its ability to provide good contrast [29,30]. However, the calculation of the radiation dose in the generalized model does not accurately segment the different brain regions of rats, and it is not possible to calculate the dose distribution among them. Therefore, we constructed a digital rat model with 16 independent brain regions and used the FDTD method to calculate the distribution of the average electric field strengths among the different brain regions under EMP radiation with different peak field strengths (0 kV/m, 100 kV/m, and 400 kV/m). The simulation results of the EMP radiation doses show that the electric field strengths in the rat brains under EMP irradiation increased with increases in the peak field strengths and that the strengths of the electric field in the rat brains increased from the dorsal to the ventral side at the same peak field strength.

Previous studies on BBB permeability in experimental animals after exposure to EMP radiation have indicated that BBB permeability exhibits temporal variations. For example, Zhou et al. [7] found that BBB permeability to horseradish peroxidase (HRP) in rats after EMP radiation (electric field strength = 400 kV/m; number of pulses = 400; repetition frequency = 1 Hz) increased within 1 h–12 h and returned to the normal level at 24 h. Zhou et al. [31] found that EMP radiation (electric field strength = 400 kV/m; number of pulses = 200; repetition frequency = 1 Hz) could cause an increase in the level and activity of collagenase in the BBB, leading to an increase in BBB permeability. These changes occurred over time and were most obvious 6 h after EMP radiation. Ding et al. [2,5] found that after EMP radiation (electric field strength = 200 kV/m; number of pulses = 200~400; repetition frequency = 1 Hz), the leakage of cerebral capillaries in rats occurred 1 h after EMP radiation, reached a peak at 3 h, and basically returned to normal at 12 h. The permeability of the rat BBB to Evans blue (EB) started to increase at 0.5 h, gradually increased from 1 h to 3 h, and then declined at 6 h. Lanthanum nitrate ions started to penetrate into the tight junctions and the basement membrane layer at 1 h, entered into the brain parenchyma at 3 h, and then the amount of extravasated lanthanum nitrate ions decreased after 6 h. Gao et al. [8] exposed rats to a single UWB-EMP at different electric field strengths (electric field strength = 50, 200, or 400 kV/m; number of pulses = 20,000; repetition frequency = 10 Hz). The BBB was examined using albumin immunohistochemistry and Evans blue staining at different time points (0.5 h, 3 h, 6 h, and 24 h) after exposure. They found that the BBB permeability of rats exposed to UWB-EMP increased immediately after the UWB-EMP treatment, reached its peak at 3 h–6 h after UWB-EMP radiation, and returned to the pre-exposure level at 24 h. The study indicated that the UWB-EMP-induced BBB opening was reversible. The results of our study are consistent with those of previous studies. Although the existing studies have qualitatively described the changes in BBB permeability over time after EMP radiation, studies using quantitative physiological parameters as the evaluation standard of BBB permeability and revealing changes in BBB permeability over time in various brain regions after EMP radiation have not been reported. Our study also found that the effects of EMP on the BBB changed over time. In our study, 3 h after EMP radiation (peak electric field strength = 400 kV/m; repetition frequency = 1 Hz; number of pulses = 1000), the ΔK^trans^ was significantly elevated in the cerebellum, orbital cortex, insular cortex, auditory cortex, striatum, and hippocampus. At the same time, the Δv_e_ was significantly elevated in the striatum. At 24 h after EMP radiation, BBB permeability was restored in all brain regions of the rats. The above results suggest that 400 kV/m EMP radiation can induce a reversible increase in the permeability of the BBB in rats.

Previous studies have shown that there were differences in the degree of BBB permeability alteration in various brain regions after EMP radiation. For example, Lin et al. [6] exposed mice to a field strength of 600 kV/m with 1000 pulses per day for two weeks and explored the effects of EMP radiation oxidative stress, microglial polarization, and blood–brain barrier integrity in the cerebral cortex, CA1, and CA3 regions of mouse brains. However, the above studies only qualitatively showed differences in BBB permeability alterations in different brain regions after EMP radiation. Therefore, based on quantitative DCE-MRI, we quantitatively revealed the spatial distribution pattern of BBB permeability alterations across the whole brain for the first time in this study. We compared the spatial distribution patterns of BBB permeability alterations in rats at the whole-brain scale after EMP radiation with different electric field strengths (0 kV/m, 100 kV/m, and 400 kV/m). The results did not show changes in the spatial distribution of BBB permeability after EMP radiation. The difference between our current work and previous studies may be attributed to the differences in the physical parameters of EMP radiation or differences in the research methods used (we used DCE-MRI, while previous studies [6] have used EB staining).

The dose–effect relationship between the EMP radiation dose and its induced biological effects has always been an important element in the study of EMP biological effects. Previous studies have preliminarily shown that changes in BBB permeability are related to the electric field strength. For example, Gao et al. [8] exposed rats to a single ultra-wide band electromagnetic pulse (UWB-EMP) at different electric field strengths (electric field strength = 50, 200, or 400 kV/m; number of pulses = 20,000; repetition frequency = 10 Hz). The BBB was examined using albumin immunohistochemistry and Evans blue staining, and they found that UWB-EMP-induced BBB permeability was field strength-dependent. However, previous studies based on histopathological methods have lacked quantitative analysis, and a quantitative correlation between the EMP radiation dose and BBB permeability has not been reported. In this study, we analyzed the Pearson correlation between the ΔK^trans^ and Δv_e_ values and the regional electric field strength in each sensitive brain region. The results suggest that there is a significant positive relationship between BBB permeability and electric field strength in rat brains within a range of 0 to 400 kV/m 3 h after EMP radiation. This result shows a certain degree of consistency with previous studies [2,8]. However, when the external electric field strength was 400 kV/m, the positive relationship between the regional electric field strength and BBB permeability across different brain regions was not observed. A possible reason for this is that BBB permeability may reach a threshold. An increase in the field strength beyond this point will no longer proportionally enhance the permeability. Another possible reason could be that the differences in tissue specificity mask the regional linear trend. However, these explanations need to be verified through further exploration.

The opening of the BBB induced by EMP found in this study may be achieved through a complex interplay of biological processes. One possible mechanism is associated with the activation of microglia-driven neuroinflammation. It has been reported that EMP radiation can cause increased levels of oxidative stress. Such a change in oxidative stress triggers microglial polarization to the M1 state, leading to neuroinflammation and disruption of the BBB by the pro-inflammatory response of the microglia [6]. Another possible mechanism is related to the disruption of tight junction (TJ) proteins, which are crucial for maintaining the integrity of the BBB. As indicated in previous studies [5,6,7,8,31,32,33], after EMP radiation, alterations in the expressions and locations of certain TJ proteins (such as ZO-1 and claudin-5) were involved in EMP-induced BBB permeability. For example, Ding et al. [5] found that BBB permeability increased concurrently with the abnormal expression and distribution of some TJ proteins (ZO-1) after EMP radiation. Specifically, the expression level of ZO-1 decreased significantly and its location changed 1 h and 3 h after EMP radiation, and then began to recover at 6 h, which is consistent with the changes in BBB permeability. These results suggest that changes in tight junction (TJ) proteins may play an important role in BBB dysfunction after EMP radiation, and the recovery of TJ proteins over time explains the reversible changes in BBB permeability induced by EMP.

Over the past several decades, various methods have been developed to overcome issues related to BBB permeability. These include local delivery strategies that bypass the BBB and physical mechanisms to increase its permeability [8]. The former approach is highly invasive and mainly refers to convection-enhanced delivery (CED), which utilizes positive pressure to directly inject anti-cancer drugs into brain tumors, thereby bypassing the BBB [34]. The latter approach has been reported to reversibly open the BBB with relatively low invasiveness [35]. Reversibly inducing an increase in BBB permeability using EMP is one of the latter methods. For the clinical application of EMP, it is crucial to evaluate the relationship between the biological effects and EMP parameters, such as the electric field strength. However, studies regarding the impact of EMP on BBB permeability remain inadequate. DCE-MRI has been extensively employed in the research of BBB permeability due to its advantages in quantitative analysis and dynamic longitudinal intra-subject tracking. For example, Wu et al. utilized DCE-MRI to investigate the impact of microbubbles combined with focused ultrasound on BBB permeability. Nevertheless, previous studies on the effect of EMP on BBB permeability have predominantly relied on histopathological approaches. For example, Gao et al. employed albumin immunohistochemistry and Evans blue staining to examine BBB permeability. However, previous studies based on histopathological methods have lacked quantitative analysis and longitudinal intra-subject monitoring. Compared with histology-based methods, DCE-MRI is more suitable for use in longitudinal studies, allowing for the repeated scanning of a rat without sacrifice. A series of imaging throughout the entire experiment can offer higher statistical power. In this study, taking advantage of DCE-MRI, we found that 400 kV/m EMP radiation could induce a transient BBB opening that could recover 24 h after the exposure. Compared to the findings based on histopathological methods, our results provided more direct evidence of the reversibility of BBB opening after EMP radiation.

The current study still has several limitations. First, the DCE-MRI acquisition was conducted 1 d before and 3 h and 24 h after exposure to EMP radiation. The advantage of DCE-MRI in investigating the temporal variation in BBB permeability after EMP radiation warrants further exploration. In the future, DCE-MRI acquisition could be performed at additional time points to facilitate a more comprehensive understanding of the detailed kinetic progression of BBB opening following EMP radiation. Second, though DCE-MRI has advantages for longitudinal investigation of BBB permeability, it has technical limitations. One of them was its limited ability to assess BBB opening to different-sized molecules. DCE-MRI relies on a contrast agent that is usually of a certain size. The results found in a study using Gd-DTPA (0.9 kDa) may not be applied to molecules with different weights (e.g., albumin, approximately 66 kDa, or immunoglobulins, approximately 150 kDa). To overcome this limitation, applying DCE-MRI with multi-size gadolinium-based nanoparticles or integrating DCE-MRI with other modality imaging techniques, such as positron emission computed tomography (PET), could facilitate size-selective permeability mapping. Another limitation of DCE-MRI was its indirect assessment of the BBB function via contrast agents. In contrast, histopathological methods enable the analysis of BBB alterations at cellular and molecular levels. In the future, following DCE-MRI detection, histopathological approaches could be conducted to further interpret the mechanism of BBB permeability changes. Third, in this study, only head motion control was implemented to prevent the motion artifact in DCE-MRI. In future research, motion correction during image processing could be implemented to further reduce the influence of motion on DCE-MRI images. Finally, this study mainly focused on the dose–effect relationship between the electric field strength of EMP and BBB permeability. In the future, multifactorial experimental designs, systematically varying parameters such as the number of pulses and repetition frequency of EMP radiation, could be adopted to further investigate the relationship between these physical parameters of the EMP and the permeability of the BBB.

## 5. Conclusions

In conclusion, 400 kV/m EMP radiation can induce a transient BBB opening that can be recovered 24 h after the exposure. However, no spatial differences in the BBB permeability were observed in rats’ brains using DCE-MRI. Furthermore, there is a significant positive linear relationship between the BBB permeability of the rat brains and the regional electric field strength of EMP radiation within an external electric field strength range of 0 to 400 kV/m at 3 h after EMP radiation. This study demonstrated the promising potential of employing EMP for transient openings in the BBB, which could facilitate clinical pharmacological interventions via drug delivery into the brain.

## Data Availability

The experimental data used to support the findings of this study are included in the article. Further inquiries can be directed to the corresponding authors.

## Supplementary Materials

The following supporting information can be downloaded at https://www.mdpi.com/article/10.3390/brainsci15060577/s1, Figure S1: Results of the linear regression analysis between the regional electric field strength E in the rat brain and the ΔK^trans^ value and Δv_e_ value 3 h after 400 kV/m EMP radiation.

## Abbreviations

The following abbreviations are used in this manuscript:

BBB	Blood–brain barrier
CED	Convection-enhanced delivery
DCE-MRI	Dynamic contrast-enhanced magnetic resonance imaging
EMP	Electromagnetic pulse
EB	Evans blue
FA	Flip angle
FDTD	Finite difference time domain
FLASH	Fast low-angle shot
HRP	Horseradish peroxidase
K^trans^	Volume transfer constant
PET	Positron emission computed tomography
ROI	Region of interest
TE	Echo time
TR	Repetition time
UWB-EMP	Ultra-wide band electromagnetic pulse
v_e_	Extravascular extracellular volume fraction
ΔK^trans^	Change in K^trans^
Δv_e_	Change in v_e_
