# Supplementary material for: Alterations in the Temporal Variation and Spatial Distribution of Blood–Brain Barrier Permeability Following Electromagnetic Pulse Radiation: A Study Based on Dynamic Contrast-Enhanced MRI
Source: Brain Sci. 2025 May 27;15(6):577. doi: 10.3390/brainsci15060577 (PMC12191220; doi:10.3390/brainsci15060577)
Supplement: Supplementary file 1 [file brainsci-15-00577-s001.zip › brainsci-3626611-supplementary.pdf]

Supplementary Information

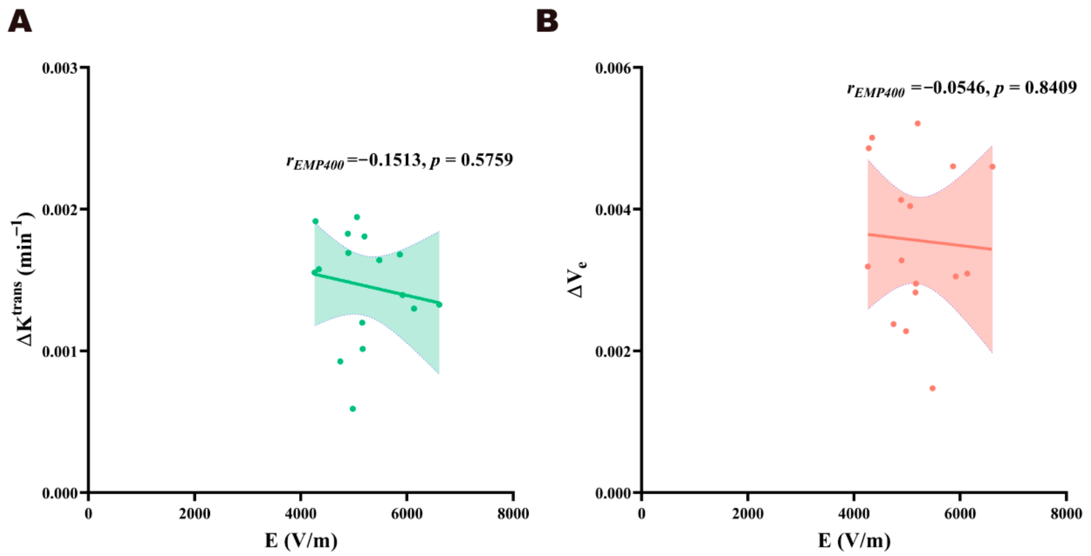

Figure S1. Results of the linear regression analysis between the regional electric field strength in the rat brain E in the rat brain and the ΔK^{trans} value and Δv_e value 3 h after 400 kV/m EMP radiation. **(A)** The linear relationship between the E value and the corresponding ΔK^{trans} value within the EMP400 group ($r = -0.1513$, $p = 0.5759$). **(B)** The linear relationship between the E and the corresponding Δv_e value within the EMP400 group ($r = -0.0546$, $p = 0.8409$). Pearson's correlation analysis was carried out to investigate the relationship between the E values and the corresponding ΔK^{trans} and Δv_e values of all brain regions in the rats. The linear regression equations were obtained by linear regression analysis. E represents the regional electric field strength in the rat brain.
